# Mitochondrial ROS signalling requires uninterrupted electron flow and is lost during ageing in flies

**DOI:** 10.1007/s11357-022-00555-x

**Published:** 2022-03-30

**Authors:** Charlotte Graham, Rhoda Stefanatos, Angeline E. H. Yek, Ruth V. Spriggs, Samantha H. Y. Loh, Alejandro Huerta Uribe, Tong Zhang, L. Miguel Martins, Oliver D. K. Maddocks, Filippo Scialo, Alberto Sanz

**Affiliations:** 1 Institute for Cell and Molecular Biosciences, Newcastle University Institute for Ageing, Newcastle University, Campus for Ageing and Vitality, Newcastle upon Tyne, NE4 5PL UK; 2 Faculty of Medical Sciences, Wellcome Centre for Mitochondrial Research, Biosciences Institute, Newcastle University, Newcastle upon Tyne, NE4 5PL UK; 3 MRC Toxicology Unit, University of Cambridge, Cambridge, CB2 1QR UK; 4 Hearing Sciences, School of Medicine, University of Nottingham, Nottingham, NG7 2UH UK; 5 Institute of Cancer Sciences, Wolfson Wohl Cancer Research Centre, University of Glasgow, Glasgow, G61 1QH UK; 6 Present Address: Novartis Institutes for BioMedical Research, Shanghai, 201203 China; 7 Institute of Molecular, Cell and Systems Biology, College of Medical, Veterinary and Life Sciences, University of Glasgow, Glasgow, G12 8QQ UK; 8 Dipartimento Di Scienze Mediche Traslazionali, Università Degli Studi Della Campania “Luigi Vanvitelli”, 80131 Naples, Italy

**Keywords:** Ageing, Complex I, Complex IV, *Drosophila*, Mitochondria, Reverse electron transport, Reactive oxygen species

## Abstract

Mitochondrial reactive oxygen species (mtROS) are cellular messengers essential for cellular homeostasis. In response to stress, reverse electron transport (RET) through respiratory complex I generates high levels of mtROS. Suppression of ROS production via RET (ROS-RET) reduces survival under stress, while activation of ROS-RET extends lifespan in basal conditions. Here, we demonstrate that ROS-RET signalling requires increased electron entry and uninterrupted electron flow through the electron transport chain (ETC). We find that in old fruit flies, ROS-RET is abolished when electron flux is decreased and that their mitochondria produce consistently high levels of mtROS. Finally, we demonstrate that in young flies, limiting electron exit, but not entry, from the ETC phenocopies mtROS generation observed in old individuals. Our results elucidate the mechanism by which ROS signalling is lost during ageing.

**Supplementary Information:**

The online version contains supplementary material available at 10.1007/s11357-022-00555-x.

## Introduction

Mitochondria are double-membrane organelles which produce a significant amount of cellular energy. Mitochondria are also instrumental for the synthesis of iron-sulphur clusters, nucleotides for DNA replication and the generation of metabolic intermediates required for anabolism [45]. These fundamental roles mean that mitochondrial function influences crucial processes affecting cell growth, differentiation and death by controlling senescence and apoptosis [8, 18]. A hallmark of ageing is the accumulation of damaged mitochondria that produce high levels of mitochondrial reactive oxygen species (mtROS) [19]. The negative consequences of elevated levels of ROS, i.e. loss of redox signalling and oxidative stress, are well known [43],however, it is unclear how and why this occurs. Due to the central role of mitochondria in maintaining cellular homeostasis, we anticipate that inhibition of “mitochondrial ageing” will improve healthspan and contribute to a delayed onset of diseases associated with ageing.

To understand how mitochondrial ageing occurs, we must identify the physiological processes regulated by mtROS and study how they are altered during ageing. It is therefore imperative that the connection between changes in mtROS and the accumulation of respiratory-deficient mitochondria is understood. In the past, we have shown that in response to stress, fly mitochondria produce ROS via reverse electron transport (RET) (ROS-RET) [41]. ROS-RET occurs under particular conditions, when both a highly reduced coenzyme Q (CoQ) pool and elevated proton motive force (pmf) coincide to allow RET from ubiquinol to CI [33]. Suppression of ROS-RET under stress prevents an adaptative transcriptional response and shortens survival in both fruit flies [41] and mice [9]. On the other hand, stimulation of ROS-RET in basal conditions, through expression of an alternative NADH dehydrogenase, extends the lifespan of *Drosophila* [40, 40]. This indicates the existence of a mitochondrial redox signalling pathway that acts to regulate lifespan under both basal and stress conditions.

Stress adaptation in flies relies upon ROS-RET, which requires perfectly coupled mitochondria. We hypothesised that ROS-RET signalling would be affected by age-associated mitochondrial alterations, limiting stress adaptation in old individuals. Here, we resolve the mechanism mediating ROS-RET signalling in detail. We combine high-resolution respirometry and metabolomic profiling to characterise how ROS-RET occurs in vivo. We show that ROS-RET is triggered by an increase in electron entry into the electron transport chain (ETC). Next, we demonstrate that ROS-RET signalling is lost during ageing and replaced by sustained high levels of ROS production. At the organismal level, we show that loss of ROS-RET compromises stress adaptation in old individuals. Finally, we dissect the ETC alterations responsible for the loss of ROS-RET during ageing. We find that reducing electron exit in young individuals suppresses ROS-RET signalling resulting in an acute increase in ROS production upstream of complex IV (CIV). Like in old mitochondria, mitochondria where CIV is depleted are unresponsive to stress stimuli and phenocopy the mitochondrial ageing phenotype observed in aged individuals.

## Material and methods

### Fly husbandry

Wild-type flies (white Dahomey, wDAH), RNA interference (RNAi) and GAL4 driver lines were collected and cultured as in [41]. Briefly, flies were maintained on standard media (1% *Drosophila* agar type II (Dutscher Scientific, #789,150), 1.5% sucrose (Sigma, #S27480), 3% glucose (Sigma, #16,325), 3.5% dried yeast (Dutscher Scientific, #789,093), 1.5% maise (TRS), 1% wheat (MP Biomedicals, #0,290,328,805), 1% soy (Santa Cruz Biotechnology, #Sc-215897A), 3% treacle (Bidvest, #90028S), 0.5% propionic acid (VWR, #8.00605.2500), 0.1% Nipagin (Sigma, #H5501)), collected using CO_2_ anaesthesia within 24 h of eclosion and maintained at a density of 20 flies per vial at 25 °C. Female flies 2–5 days old, unless otherwise stated, were used for all experiments. UAS-*levy*-RNAi (CG17280, 101,523) and UAS-*ND*-75-RNAi (CG2286, 100,733) were obtained from the Vienna Drosophila Resource Centre (VDRC), while daughterless-GAL4 (daGAL4) was acquired from the Bloomington Drosophila Stock Centre (BDSC). ETC inhibitors, rotenone (Santa Cruz Biotechnology, #Sc-203242) and cyanide (Sigma, #60,178), were dissolved in ethanol and water and added to the fly food at a final concentration of 600 µM and 18 mM, respectively.

### Thermal stress and lifespan studies

Thermal stress (TS) was induced by transferring flies cultured at 25 to 32 °C for 3–4 h. Except for lifespan experiments, flies were used immediately after TS for all experiments. For lifespan experiments, flies were TS three times per week for 4 h in either the presence or absence of rotenone and then returned to 25 °C (in the absence of rotenone).

### Measurement of ROS levels in Drosophila brains

ROS were measured in individual fly brains as described in [41]. Briefly, 2′,7 ′-dichlorofluorescein (H_2_DCF) was used to detect total levels of ROS. Brains were dissected in phosphate-buffered saline (PBS). Following dissection, brains were incubated in 30 μM H_2_DCF for 10 min, washed three times with 1X PBS, and imaged immediately. Images were acquired using a LSM510 confocal microscope (Zeiss) equipped with a 10 × 0.3 NA objective as z stacks throughout the sample, using a 488-nm line of an Argon laser to excite H_2_DCF. The total average fluorescence intensity of each brain was quantified using ImageJ.

### Measurement of mitochondrial oxygen consumption

Mitochondrial oxygen consumption was assessed via high-resolution respirometry using an Oxygraph 2-K (Oroboros Instruments) as described in [40, 40]. Briefly, whole flies (10–20) or fly heads (20–30) were homogenised in isolation buffer (250 mM sucrose, 5 mM Tris–HCl, 2 mM EGTA, pH 7.4). Homogenates were diluted 20 times in assay buffer (120 mM KCl, 5 mM KH_2_PO_4_, 3 mM HEPES, 1 mM EGTA, 0.5 mM MgCl_2_, 0.2% (w.v.) BSA, pH 7.2) for analysis. Mitochondrial activity was initiated through the addition of pyruvate (5 mM) and proline (5 mM) followed by ADP (1 mM) to initiate state 3. Oxygen consumption was quantified using Oroboros DatLab 5.0 software with oxygen flux raw values normalised to the amount of fly protein. Final values were expressed as picomoles of oxygen per min^−1^ per mg^−1^.

### Metabolomic analysis by liquid chromatography-mass spectrometry (LC–MS)

In total, 20 heads per sample (three independent samples) were homogenised in ice-cold lysis solvent (methanol 50%, HPLC grade acetonitrile 30%, water 20%) and centrifuged at 13,000–15,000 rpm for 15 min at 4 °C. The supernatant was stored at − 80 °C. Before analysis, samples were transferred to LC–MS vials. LC–MS measurements and related data analysis were performed as described previously [20], using a ZIC-pHILIC analytical column. LC–MS raw data were converted into mzML files using ProteoWizard. MZMine 2.10 was used for peak extraction and sample alignment. For global metabolomics, metabolites were analysed with the help of MetaboAnalyst [46]. A CSV file containing the intensity values of the peaks of all the metabolites detected was uploaded into MetaboAnalyst, and the statistical analysis tool was selected. Data were analysed with the following options: data filtering (none), data transformation (log), and data scaling (auto). Samples grouped by treatment were analysed by ANOVA, and data were ranked according to FDR values (*p* < 0.05). Afterwards, selected metabolites were used for enrichment (input type: KEEG ID/metabolites,feature type: KEEG) and pathway analysis (visualisation method: scatter plot,enrichment method: hypergeometric test; topology analysis: relative-betweenness centrality; reference metabolome: Homo sapiens KEEG) using the enrichment and pathways analysis tools of MetaboAnalyst respectively with a cut-off of *p* ≤ 0.05.

### Next-generation sequencing: data acquisition and analysis

RNA was extracted from fly heads (20 heads per sample, five independent samples per condition). Heads were homogenised in TRI Reagent (Sigma) following the manufacturer's instructions. RNA was treated with DNase I (Thermo Fisher Scientific) at 37 °C for 60 min and precipitated overnight with 3 M sodium acetate and 95% ethanol. After centrifugation, pellets were dissolved in 50 µl of DNase/RNase free water. The RNA quality was confirmed using an Agilent 2100 Bioanalyzer (Agilent Technologies). Detailed experimental protocols and raw data are deposited in ArrayExpress under accession E-MTAB-7952. Briefly, next-generation sequencing data acquisition was performed using the TruSeq Stranded mRNA kit (Illumina) following the manufacturer's instructions. Raw data were acquired using an Illumina sequencer (NextSeq500) and processed using Partek Flow (Partek Inc. Missouri, USA). RNA reads were normalised using the default method (total count, add 0.0001) and aligned to Reference Index BDGP6 using STAR 2.4.d. To select transcripts that were up- or downregulated for subsequent analysis, we filtered transcripts whose fold change (FC) expression was above or below ± 1.5, respectively, discarding those whose FDR was above 5%.

### Statistical analysis

Data are shown as mean ± SEM and were analysed using GraphPad Prism 9 with either unpaired student’s *t* test or one-way ANOVA, with Dunnett’s post-test where appropriate. In addition, lifespan data were analysed using the Kaplan–Meier log-rank test. Cartoons and schematic diagrams were generated with the help of biorender.com.

## Results

### ROS-RET is activated by increased electron flow into the ETC

We and others have shown that ROS-RET requires both an increase in the redox state of the CoQ pool and a sufficiently high pmf to occur [33, 40, 39, 41]. It remains unclear, however, how these conditions would coincide in a physiologically relevant context. In mitochondria, the nature of the substrate that is oxidised (e.g. pyruvate versus fatty acids) determines where and how mtROS are generated [3, 12]. For example, increased oxidation of substrates downstream of CI, such as succinate, fatty acids, or dimethylglycine, stimulates ROS-RET [6, 12, 22]. While alterations in ROS levels reprogramme cellular metabolism [37], physiological examples of this include cellular differentiation [44] and adaptation to thermal [41] or oxidative stress [17]. Here, we dissect both the mechanism(s) that drive ROS-RET production and the downstream metabolic signals triggered by ROS-RET under TS. This type of natural stress has strong effects on reproduction and survival in insects such as fruit flies and is therefore highly relevant.

**Fig. 1:**
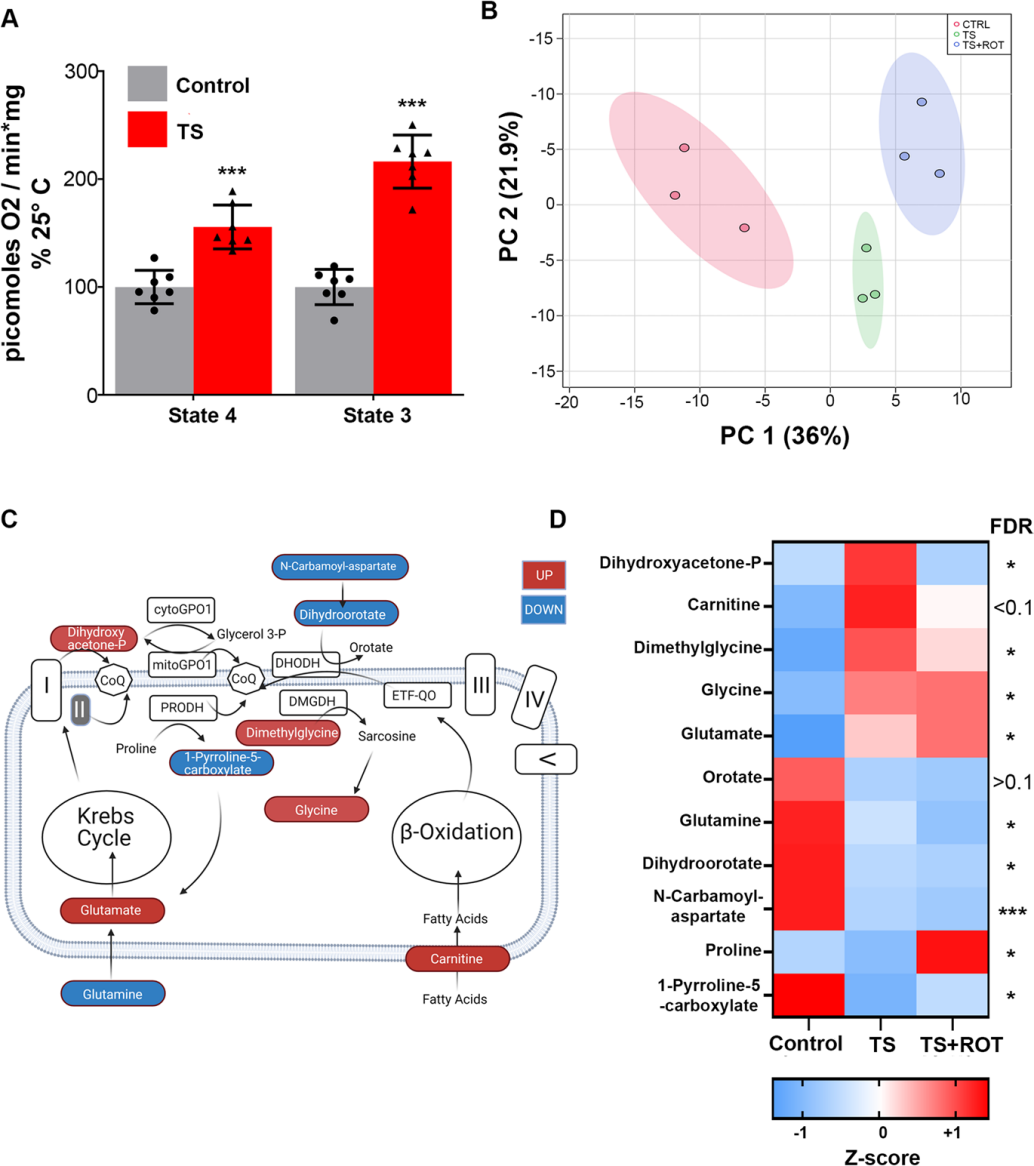
Thermal stress (TS) increases electron flow through and downstream of CI. **A** Mitochondrial oxygen consumption measured in state 4 (without ADP) and state 3 (with ADP). Data are shown as mean ± SEM. *N* = 7 per experimental group. **B** PCA analysis of the brain metabolome of Control flies and TS flies with (TS + ROT) and without rotenone (TS). **C** Schematic representation highlighting key metabolites which show alterations in response to ROS-RET and their contribution to the reduction of ubiquinone to ubiquinol. Blue colouring indicates metabolites with decreased levels, while red indicates those with increased levels. cytoGPO1 = cytosolic glycerol-3-phosphate dehydrogenase; mtGPO1, mitochondrial glycerol-3-phosphate dehydrogenase; PRODH, proline dehydrogenase; DHODH, dihydroorotate dehydrogenase; DMGDH, dimethylglycine dehydrogenase; ETF-QO, electron-transferring-flavoprotein dehydrogenase. **D** Heat map of metabolites potentially contributing to the generation of ROS-RET in response to TS. NS, not significant, *p < 0.05, **p < 0.01, ***p < 0.001. *N* = 3 in all experiments unless otherwise stated

We have previously shown that 3 h of TS induces ROS-RET production in the fly brain. To further understand how ROS-RET occurs, we TS flies and measured mitochondrial oxygen consumption. We found a significant increase in state 4 (non-phosphorylating) and state 3 (phosphorylating) respiration (Fig. 1A), indicative of increased electron flow through the ETC. We believe that this increase in electron entry into the ETC is instrumental for ROS-RET production. In line with this, we have shown that reducing electron flow by depleting either CI or complex II (CII), using RNAi interference, abolishes ROS-RET in flies [41]. Similarly, depletion of CI, by knocking-out Ndufs2, specifically in cells of the carotid body prevents generation of ROS-RET and the adaptive response to hypoxemia in mice [10], whereas inhibition of electron flow through CI or CII, using rotenone or malonate, respectively, in mouse hearts diminished damage resulting from ischemia–reperfusion [6]. This suggests that the role of CI and CII in initiating ROS-RET production is conserved across evolution.

Next, we performed unbiased metabolomic profiling on heads from flies TS (Fig. S1A). We also profiled flies fed with rotenone to distinguish between metabolic changes triggered by ROS-RET production and those as a response to heat. Rotenone blocks RET from ubiquinol to CI and is considered the gold standard for demonstrating the occurrence of ROS-RET [6, 3, 28, 39, 40]. Unsupervised principal component analysis clearly separated the three experimental groups (Fig. 1B), suggesting that specific metabolic changes occur upstream and downstream of ROS-RET production, with potential implications for signalling and stress adaptation. We began by studying the metabolites required to trigger ROS-RET. We selected those metabolites that were significantly altered in TS flies. Pathway and enrichment analysis revealed alterations in the metabolism of amino acids such as serine and glutamate (Fig. S1B-D). This is in line with previous observations describing that, under stress, glutamate and serine are rerouted to the ETC [48, 49] where they contribute to the reduction of ubiquinone to ubiquinol.

Manual inspection revealed significant changes in the levels of 9 metabolites with the potential to reduce ubiquinone to ubiquinol (Fig. 1C-D and S1E). Firstly, we observed a decrease in the levels of glutamine and a concomitant increase in the levels of glutamate (Fig. 1C-D and S1E). This has been described as an alternative way to feed the ETC when pyruvate oxidation is totally or partially interrupted [48]. Here, we explain the former, as an additional way to address the increase in energy demand by boosting amino acid-oxidation via the Krebs Cycle-ETC. Another way of obtaining glutamate and subsequently reductive power for the ETC via the Krebs cycle is proline oxidation [11]. Supporting the activation of this route, we detected a deep decline in the product of oxidation of proline by the mitochondrial proline dehydrogenase: 1-pyrroline-5-carboxylate (Fig. 1D and S1E). The former is further converted into glutamate by the pyrroline-5-carboxylate dehydrogenase [30]. Our interpretation of the metabolomic changes detected is further supported by data showing that insect mitochondria oxidise both proline and glycerol-3-phoshate to boost ATP production during flight [36] and when exposed to temperatures above 30 °C [15]. Accordingly, levels of dihydroxyacetone-phosphate, the product of oxidation of glycerol-3-phosphate, were increased in response to TS (Fig. 1D and Fig. S1E). It is important to note that we observed a modest increase in the levels of proline and lack of increase in the levels of dihydroxyacetone phosphate in rotenone-fed flies (Fig. 1D and S1E). We reckon that the former is to prevent NAD + depletion. Since NADH is generated from proline and glyceraldehyde 3-phosphate oxidation, to feed the ETC, and rotenone feeding prevents reoxidation of NADH (by inhibiting CI), it is plausible that in rotenone-fed flies, the oxidation of both proline and glyceraldehyde 3-phosphate is paused to prevent the catastrophic consequences of NAD + depletion. Supporting reoxidation of NADH as the problem in rotenone-fed flies, we observed a strong increase in the levels of lactate (see below for a more detailed explanation).

Secondly, we also observed evidence indicating increased oxidation of dihydroorotate, choline, and fatty acids. These three processes introduce electrons into the CoQ pool downstream of CI and CII and increased fat usage has been shown to trigger ROS-RET in mouse cells [12]. Both N-carbamoyl-aspartate and dihydroorotate levels were strongly depleted in TS flies (Fig. 1C-D and S1E). N-carbamoyl-aspartate is the precursor of dihydroorotate which is oxidised by mitochondrial dihydroorotate dehydrogenase to produce orotate, a precursor required for the synthesis of pyrimidines [23]. Supporting elevated mitochondrial oxidation of choline, we found an increase in the levels of two downstream products: dimethylglycine and glycine (Fig. 1D and S1E). Interestingly, dimethylglycine initiates ROS-RET when fed to isolated mitochondria [22]. However, the support for elevated fatty acid oxidation was modest since we only observed a small increase in the TS group that was not fed with rotenone (Fig. 1D and S1E).

All in all, our results offer indirect evidence indicating that TS activates ROS-RET production increasing mitochondrial respiration with an increased entry of electrons through both CI/II as well as other dehydrogenases. These dehydrogenases are not usually considered significant contributors to ATP production, but they have been reported to participate in ROS generation [11], 12, 22, 25].

### ROS-RET reroutes glycolytic intermediates to the pentose phosphate pathway (PPP) but does not induce a short-term transcriptomic response

**Fig. 2:**
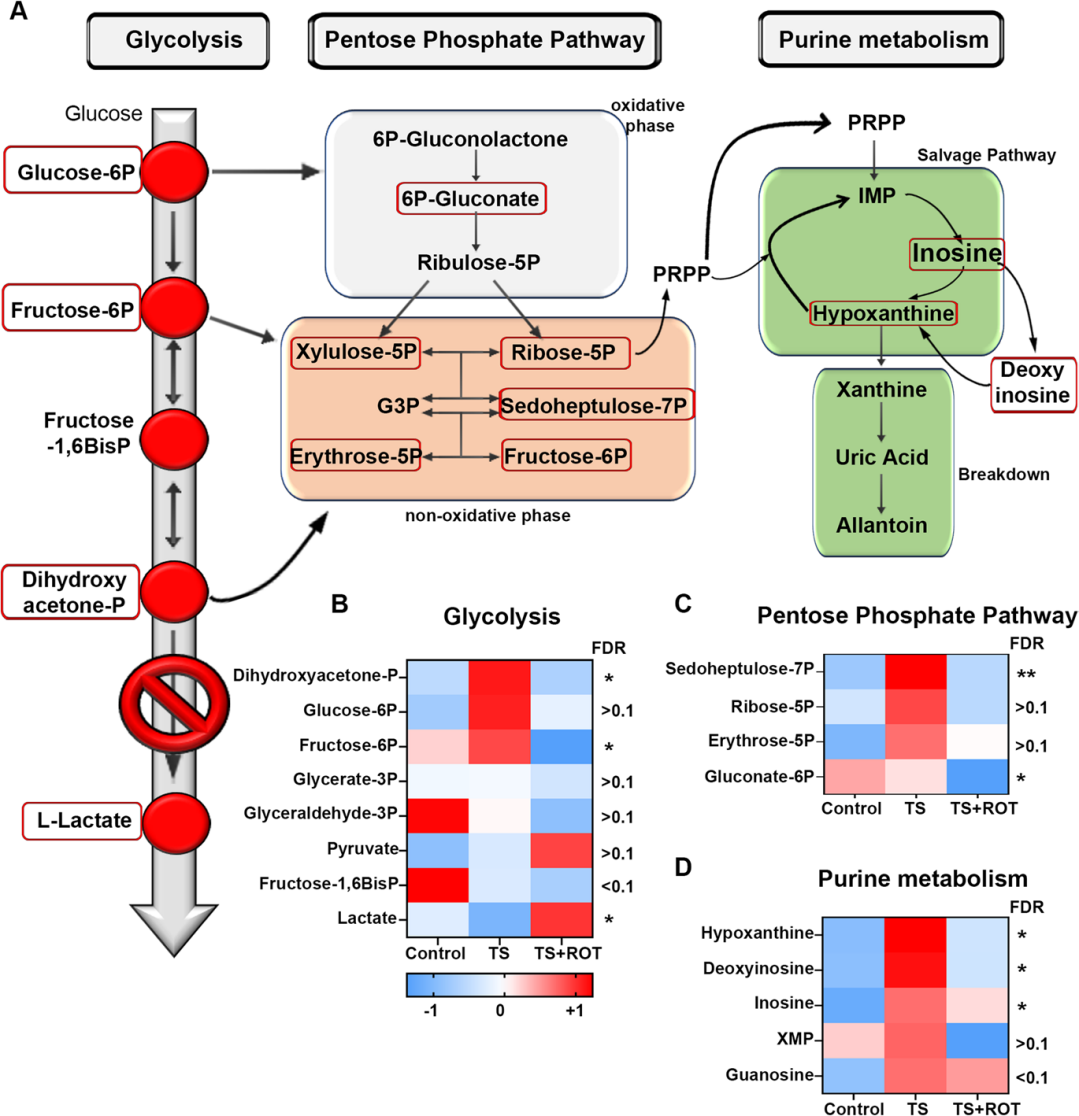
ROS-RET results in the redirection of glycolytic intermediates to the pentose phosphate pathway (PPP) to produce NADPH and nucleotide precursors. **A** Schematic representation of the biological pathways affected by ROS-RET signalling. Metabolites significantly altered by ROS are indicated in red boxes. **C**–**D** Heat maps of glycolytic, PPP and purine biosynthesis metabolites identified in the fly brain in control flies and TS flies with (TS + ROT) and without rotenone (TS). NS, not significant, *p < 0.05, **p < 0.01, ***p < 0.001. *N* = 3 in all experiments unless otherwise stated

We next investigated the metabolic changes initiated downstream of ROS-RET by selecting metabolites that were altered (up or down) in response to TS but had their levels reverted by rotenone feeding (Fig. S1A). Enrichment and pathway analysis identified PPP, glycolysis, purine, and glutathione metabolism as the main metabolic routes modified by ROS-RET signalling (Fig. S2A-C). Our metabolomic analysis suggests that ROS-RET inhibits glycolysis, resulting in the redirection of glycolytic intermediates to PPP, allowing maintenance of NADPH levels (depleted during TS) and production of nucleotide precursors (Fig. 2B-D). Accordingly, we observed upregulation of three glycolytic intermediates (dihydroxyacetone phosphate, fructose 6-phosphate, and glucose 6-phosphate), three PPP metabolites (sedoheptulose 7-phosphate, erythrose 5-phosphate, and ribose 5-phosphate), and three components of the salvage purine synthesis pathway (hypoxanthine, inosine, and deoxyinosine) only in those TS flies that were not fed with rotenone (Fig. 2D and S2F).

**Fig. 3:**
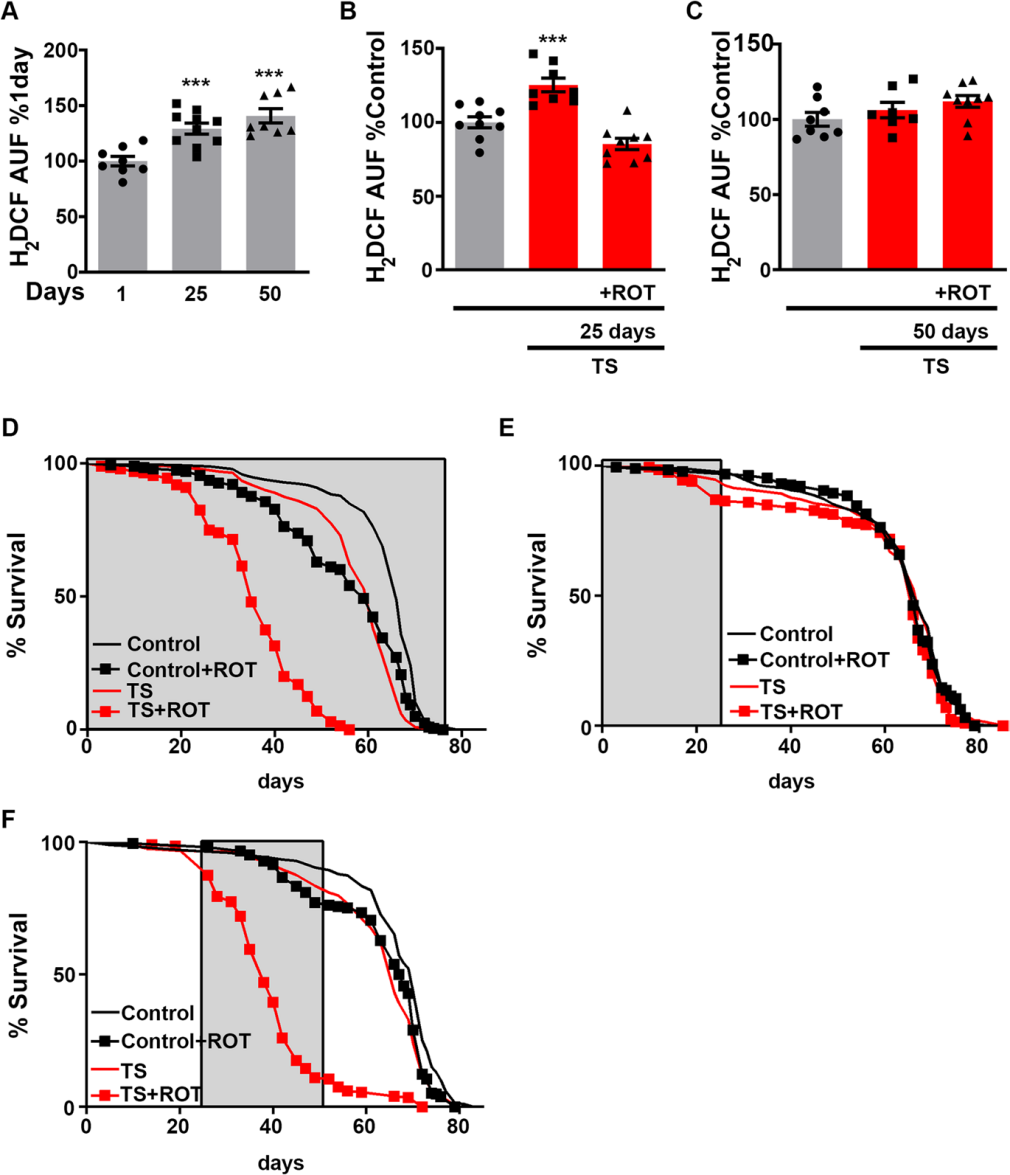
Old mitochondria continually produce high levels of ROS and are unable to activate ROS-RET signalling in response to stress. **A** ROS levels in brains of young (1 day), middle-aged (25 days) and old-aged flies (50 days). *N* = 8–10 per experimental group. **B** ROS levels in brains of middle-aged flies TS in the presence (TS + ROT) or absence of rotenone (TS). *N* = 8–9 per experimental group. **C** ROS levels in brains of old aged flies under TS in the presence of rotenone (TS + ROT) and without rotenone (TS). *N* = 7–9 per experimental group. **D** Lifespan of control flies (black line), control flies fed with rotenone 3 times a week (black squares, control + ROT), TS flies three times per week (red line, TS) and finally TS flies three times a week in the presence of rotenone (red squares, TS + ROT). Treatments were performed for the duration of fly lifespan. *N* = 199–203 per experimental group. **E** As in D, but with treatments administered only between days 1 and 25 of fly lifespan. *N* = 188–204 per experimental group. **F** Also, as in D, but with treatments administered from day 25 to Day 50. *N* = 199–210 per experimental group. **D**–**E** Grey shadow indicates duration of treatments. **A**–**C** Data are shown as mean ± SEM. ***p < 0.001

As it can be observed in Fig. 2A, the glycolytic intermediates in TS flies are those that can be rerouted into the PPP to produce reductive and anabolic power in the form of NADPH [17]. NADPH is essential for anabolic reactions and provides reductive power to antioxidant enzymes through the reoxidation of glutathione and thioredoxin. Both functions are essential under TS, where oxidative stress is increased, and anabolic power is needed to synthesise components of the anti-stress response [13, 14]. Our results suggest that ROS-RET is required for the maintenance of NADPH levels under TS, and when ROS-RET is prevented, NADPH is quickly depleted. Accordingly, NADPH levels were below the limit of detection in the brains of TS flies in the presence of rotenone (Fig. S2D and S2F). In further support of our hypothesis, levels of gluconate 6-phosphate which is instrumental for the synthesis of NADPH were strongly depleted only in those flies where ROS-RET was inhibited with rotenone, whereas TS flies able to produce ROS-RET maintained the concentration of gluconate 6-phosphate at the levels of controls (Fig. 2C and S2F). NADPH depletion was not caused by an increase in oxidative stress due to rotenone since the feeding of the former resulted in less mtROS production under TS as shown previously [41] and confirmed here (Fig. 3B). Correlating with low levels of mtROS, we observed an increase in the levels of reduced (GSH) and a decrease in the levels of oxidised glutathione (GSSG) (Fig. S2D and S2F) because of rotenone, further supporting a lack of oxidative stress in these flies.

PPP metabolites of the non-oxidative phase were only elevated in TS flies that were able to perform ROS-RET (Fig. 2C and S2F). Similarly, we also found that upregulation of metabolites involved in purine biosynthesis was suppressed when ROS-RET was inhibited by rotenone feeding (Fig. 2D and S2F). Altogether, these observations suggest that ROS-RET is required for the activation of NADPH synthesis via the rerouting of glycolytic intermediates to PPP. Purines are synthesised from ribose-5-phosphate provided by the non-oxidative phase of PPP and are required for the synthesis of nucleotides to display a long-term antistress response. In the past, we have shown that suppressing ROS-RET by expressing an alternative oxidase abolishes the transcriptional response to TS [41], and here we provide a mechanistic explanation of how this occurs at the metabolic level. Of note, we observed upregulation of pyrimidine biosynthesis that seems to be independent of ROS-RET (see Fig. 1D and S1E and section above). A similar “glycolysis-to-PPP-purine synthesis rerouting” has been reported in glioma stem cells for adaptation to acidotic stress [47] as well as in human keratinocytes and mouse macrophages exposed to increasing concentrations of H_2_O_2_ [17] or lipopolysaccharide (LPS) [4]. The LPS study is particularly relevant as increased ROS levels after LPS is a result of ROS-RET, which is suppressed in the presence of rotenone [24], as occurs under TS.

In the past, we have shown that the long-term transcriptomic response to TS requires ROS-RET signalling [41]. Here, we have analysed the brain specific transcriptome to investigate if manipulation of ROS-RET causes changes at the transcriptomic level in addition to the metabolomic level. As expected, we found upregulation of anti-stress pathways, such as the heat-shock response [42]. However, interruption of ROS-RET, via rotenone feeding did not result in any significant transcriptomic changes (Fig. S2E), and we found only one gene differentially regulated in the brains of TS flies fed with rotenone. This is like what has been described in human cells exposed to H_2_O_2_ and UV radiation, where a short-term metabolomic response occurs before any transcriptional changes [17]. In summary, our results indicate that ROS-RET elicits an acute metabolic response to TS.

### Ageing is characterised by consistently high levels of mtROS and the loss of ROS-RET signalling

High-resolution respirometry and metabolomic profiling showed that ROS-RET relies on increased mitochondrial oxygen consumption. Accordingly, we and others have demonstrated that interrupting electron flow by depleting subunits of CI or CII prevents ROS-RET signalling [6, 10, 40, 39, 41]. Since ageing is characterised by the decrease of mitochondrial respiration [34, 35, 40, 39], we anticipated that ROS-RET would be altered in old individuals. To test our hypothesis, we measured ROS levels in the brain of young (~ 1 day), middle-aged (~ 25 days), and old-aged flies (~ 50 days). We chose to study old- and middle-aged flies because mitochondrial oxygen consumption is decreased in the first group but not in the second [40, 40], and therefore if our hypothesis was correct ROS-RET should only be altered in old flies.

First, and in accordance with what we and others have reported [1, 7, 34, 35, 40, 39], we observed an accumulation of mtROS levels with age (Fig. 3A). We then decided to test the capacity to generate ROS-RET by exposing middle- and old-aged flies to TS, in the presence or absence of rotenone. In line with our hypothesis, ROS-RET production in response to TS was only detected in brains of flies with unaltered mitochondrial oxygen consumption, i.e., we detected activation of ROS-RET in middle- but not in old-aged flies (Fig. 3B and 3C). In old flies, levels of ROS did not change in response to TS and rotenone feeding did not alter the high levels of ROS, proving that they are not RET derived (Fig. 3C). Furthermore, rotenone treatment of flies in basal conditions led to increased mitochondrial ROS levels in young but not in old-aged flies (Fig. S3A, B). Our results show that during ageing CI stops producing ROS in response to both TS and rotenone and suggest that age-related loss of mitochondrial respiratory capacity [34, 35, 40, 39] results in the abolition of the activation of ROS-RET signalling in response to stress [40].

Finally, we examined the physiological relevance of ROS-RET loss. We reasoned that if ROS-RET was important for stress adaptation, then stress adaptation would be severely impacted during ageing when ROS-RET signalling is diminished. To test our hypothesis, we placed flies under TS for 4 h, three times a week, in either the presence or absence of rotenone. We performed three independent experiments where TS treatments were applied as follows: (a) for the duration of fly lifespan, (b) for the first 25 days of lifespan, or (c) from day 25 to day 50 (for details, see Fig. S3C). In experimental conditions (a), rotenone feeding dramatically shortened the lifespan of TS flies, whereas it only minimally affected the lifespan of flies in basal conditions. However, this differential effect was only evident when the flies were older than 20 days (Fig. 3D). Accordingly, when treatments (including rotenone) were stopped at day 25 (b), no differences in lifespan were observed (Fig. 3E). Conversely, when treatments began from middle age for 25 days (c), rotenone feeding severely shortened survival when flies were under TS (Fig. 3F). These results, along with the ROS measurements performed in middle- and old-aged flies, indicate that the ability to activate ROS-RET signalling progressively diminishes during ageing and that further suppression of ROS-RET through rotenone feeding in old individuals severely compromises survival.

### Blocking mitochondrial electron exit mimics the mtROS profile observed in old individuals

**Fig. 4:**
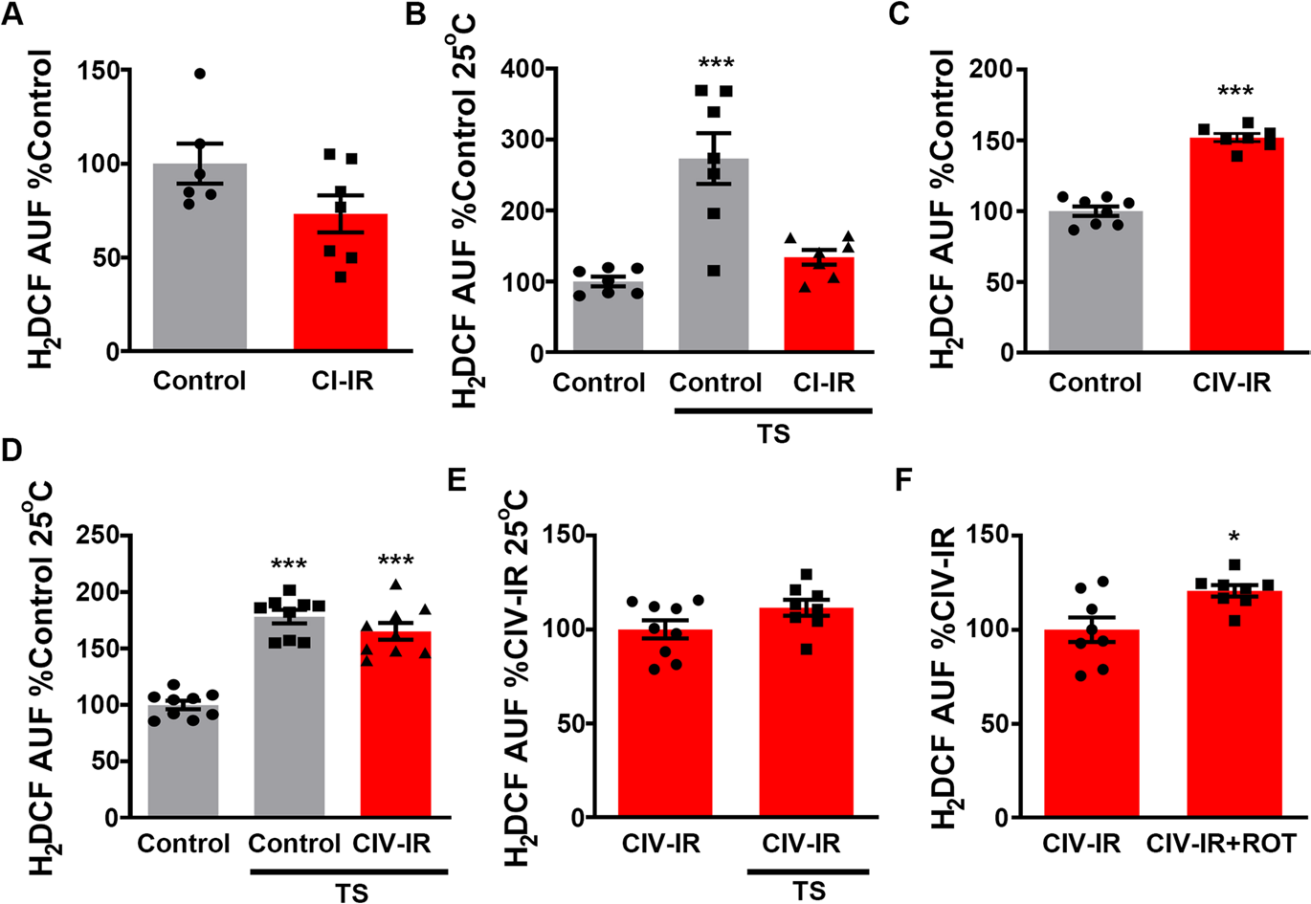
Depletion or inhibition of mitochondrial complex IV (CIV) mimics mitochondrial ROS production observed in old individuals. **A** ROS levels in brains of flies with depleted CI (CI-IR) and controls. *N* = 6–7 per experimental group. **B** ROS levels in brains of TS flies with depleted CI (CI-IR) and controls. *N* = 7 per experimental group. **C** ROS levels in brains of flies with depleted CIV (CIV-IR) and controls. *N* = 7–9 per experimental group. **D** ROS levels in brains of TS flies with depleted CIV (CIV-IR) and controls. *N* = 9 per experimental group. **E** ROS levels in brains of flies with depleted CIV levels (CIV-IR) at 25 °C or under TS. *N* = 8–9 per experimental group. **F** ROS levels brains of flies with depleted CIV (CIV-IR) in the presence (CIV-IR + ROT) or absence of rotenone (CIV-IR). *N* = 8 per experimental group. Data are shown as mean ± SEM. * *p* < 0.05, *** *p* < 0.001

To complete our study, we decided to investigate which alterations in the ETC were potentially responsible for the age-related increase in ROS levels observed in mitochondria in aged brains. We hypothesised that the accumulation of mtROS might be caused by problems in electron exit from the ETC. This would cause a build-up of electrons in the ETC, increasing the likelihood of electrons reducing oxygen to superoxide directly. This has been observed in vitro in fly mitochondria exposed to the complex IV inhibitor cyanide [35] and in mouse mitochondria from COX15 knock-out mice [9]. We predicted that this mechanism also operates in vivo during ageing. To test our hypothesis, we first decreased electron entry by depleting *ND-75,* a CI subunit, (Fig. S4A). This resulted in a ~ 50% decrease in mitochondrial respiration (Fig. S4B), analogous to the decrease observed in old flies [34, 35, 40, 39]. Depletion of *ND-75* did not affect ROS levels in basal conditions but prevented ROS-RET under TS (Fig. 4A-B), confirming our previous results [41]. This was not unexpected as reducing electron entry without blocking movement through the ETC does not increase electron leak.

We then suppressed electron exit via depletion of *levy,* a CIV subunit, (Fig. S4C). As with *ND-75*, knock-down of *levy* also decreased mitochondrial respiration by 50% (Fig. S4D). However, knock-down of *levy* increased ROS levels in the brain (Fig. 4C), indicating that decreased electron exit increases mtROS in the fly brain, as it has been shown to do in isolated mitochondria [9, 35]. Interestingly, no increase in mtROS was observed in response to TS in *levy* mutants (Fig. 4D, E). This failure to activate ROS-RET is precisely what occurs in old flies. Finally, we tested whether reducing CIV levels was sufficient to activate ROS-RET production by feeding flies with rotenone and measuring mtROS (Fig. 4F). We did not observe a decrease in the presence of rotenone and therefore were able to discard ROS-RET as the mechanism of ROS generation. To confirm the results of our genetic model, we used the specific CIV inhibitor, cyanide, to acutely block CIV. This pharmacological model avoids the uncontrolled effects resulting from long-term adaptations to genetic depletion of CIV. Cyanide treatment mimicked the genetic model by inhibiting respiration by over 50% (Fig. S4E) and strongly increasing ROS levels. As seen with genetic inhibition of CIV, rotenone treatment did not decrease ROS levels (Fig. S4F), discarding ROS-RET as a source of ROS when CIV is blocked.

## Discussion

Here, we provide mechanistic evidence of how ROS-RET signalling is activated in response to TS and describe the metabolic response it initiates. We show that ROS-RET signalling is lost during ageing and demonstrate that reducing the ability to activate ROS-RET signalling decreases the capacity of flies to adapt to TS. Finally, we dissect the mechanisms which result in continuous production of mtROS in “old mitochondria,” demonstrating that this is caused by a limitation of electron exit.

Under TS, in young mitochondria ROS-RET is activated in response to increased electron entry through CI/CII and accessory dehydrogenases that are not part of the canonical ETC. This is similar to what has been reported in other scenarios where ROS-RET is triggered [38]. For example, ROS-RET is usually associated with increased oxidation of succinate by CII, like during ischemia reperfusion [6] or hypoxia within specialised cells of the carotid body [10]. However, ROS-RET is also activated in response to increased oxidation of fatty acids in cultured mouse cells [12] and isolated mitochondria when they are fed with sn-glycerol-3-phosphate [26] or products of choline oxidation [22]. Our metabolomics analysis indicates that these processes are potentially involved in activating ROS-RET production in the brain of young flies (Fig. 1).

Our data indicate that the production of ROS-RET initiates metabolic reprogramming consisting of rerouting glycolytic intermediates to the PPP (Fig. 2). This increases NADPH production providing cells with both reductive and anabolic power. Accordingly, suppressing ROS-RET using rotenone completely depleted NADPH. Several observations support the idea that this stress response is conserved across evolution. Firstly, suppression of ROS signalling attenuates the long-term transcriptomic stress response in TS flies [41] and mice exposed to mitochondrial stress [9]. Secondly, a comparable “glycolysis-to-PPP-rerouting” response is observed in human cells in response to H_2_O_2_ treatment [17]. Similarly, LPS triggers ROS-RET in macrophages [24], and this stimulates the rerouting of glycolytic intermediates to PPP to produce NADPH [4]. A mechanism which is also used by glioma stem cells to adapt to acidosis [47] and lung cancer cells to resist high levels of oxidative stress [2]. It is not completely clear mechanistically how this rerouting occurs, but inhibition of several glycolytic enzymes through oxidation of cysteine residues is the most likely explanation based on the evidence available. In accordance, oxidation of pyruvate kinase M2 in human cells [2] and GAPDH in yeast, worms, and human cells [31] has been described alongside interruption of glycolysis and boosting of PPP. However, an independent report showed that metabolic rerouting precedes changes in the activity of glycolytic enzymes in response to increases in H_2_O_2_ [17]. We have not investigated the mechanisms by which ROS-RET activates NADPH synthesis in the fly brain in response to stress, a question which warrants further investigation. The metabolomic approach used in this study has certain limitations. Firstly, as we did not use metabolic flux analysis, we can only estimate the directionality of the metabolic changes observed, connecting them based on the previous literature. Secondly, due to the type of chromatography used -liquid- we are not detecting many metabolites that are potentially important in the context of ROS-RET but would require gas chromatography to be detected [50].

During ageing, there is an increase in the generation of mtROS [38] that will impact redox signalling. Here, we show that young mitochondria increase ROS production in response to specific stimuli such as TS and rotenone, while aged mitochondria continually produce high levels of ROS (Fig. 3). Specifically, we demonstrate that the capacity of fly brain mitochondria to produce a ROS-RET signal is progressively attenuated during ageing and that in old flies, CI cannot produce ROS in response to TS or rotenone. Further depletion of ROS-RET signalling impacts stress adaption, with old flies dying in response to TS in the presence of rotenone. We anticipate that interruption of ROS-RET signalling would also impact other processes that require mtROS, such as activation of macrophages [24], hypoxia signalling [10], and sleep regulation [16]. Although it would be tempting to suggest that some of these processes are altered during ageing due to disruptions in mitochondrial redox communication, further research, including experiments restoring ROS-RET activation in old individuals, is required.

Finally, we dissected the mechanisms that drive the overproduction of ROS in old mitochondria. We considered two possibilities which are supported by the literature, i.e., a decrease in electron entry or exit [32, 40]. Our results support that decreased electron exit is the main problem, as we observed an increase in mtROS in fly brains only when electron exit was blocked (Fig. 4). We propose that due to decreased electron flow during ageing, the ETC produces continuously high levels of ROS but is unable to produce a ROS-RET signal in response to specific stimuli such as TS. It is intriguing how and why, during ageing, CI is replaced by one or more generators inactive in younger individuals to be the main ROS generator in fly brain mitochondria. The most likely candidate to be activated during ageing is CIII, which has been shown to increase ROS when CIV is inhibited and has been described as the main generator of ROS in aged rat heart mitochondria [5, 27, 38]. However, different mitochondria may operate in different ways. For example, it has been reported that a mild knock-down of CI subunits in fly muscle increases mtROS and extends lifespan [29]. This study did not investigate neither the ROS source nor the capacity to generate ROS-RET after CI depletion. Furthermore, the authors did not investigate whether mutant flies were sensitised to stress. In the future, we will need to investigate mtROS production in different tissues and at different ages. This way, we will be able to confirm whether age-related changes occur similarly in all tissues and therefore the feasibility of implementing a general strategy to restore redox signalling or if tissue-specific interventions would be more appropriate. For example, in isolated mitochondria from the flight muscle, glycerol-3-phosphate dehydrogenase is a major generator of ROS, producing more ROS than CIII [25]. Further, it is possible that CIII’s role as a ROS generator becomes even more important during fly ageing when glycolysis is dysregulated [21]. Therefore, we may need to target different ROS generators in different tissues, although this would be a complicated route it may be the only way to generate redox interventions that impact human lifespan.

## Supplementary Information

Below is the link to the electronic supplementary material.

Supplementary file1 (DOCX 3.23 MB)

## Abbreviations

H_2_O_2_: Hydrogen peroxide
CI: Complex I
CII: Complex II
CIII: Complex III
CIV: Complex IV
CoQ: Coenzyme Q
CYA: Cyanide
ETC: Electron transport chain
PPP: Pentose phosphate pathway
pmf: Proton motive force
RET: Reverse electron transport
mtROS: Mitochondrial reactive oxygen species
ROS-RET: ROS produced via reverse electron transport
ROT: Rotenone
TCA,: Tricarboxylic acid
TS: Thermal stress
