# Supplementary material for: Mitochondrial ROS signalling requires uninterrupted electron flow and is lost during ageing in flies
Source: GeroScience. 2022 Mar 30;44(4):1961–74. doi: 10.1007/s11357-022-00555-x (PMC9616974; doi:10.1007/s11357-022-00555-x)
Supplement: Supplementary file 1 — Supplementary file1 (DOCX 3.23 MB) [file 11357_2022_555_MOESM1_ESM.docx]

**
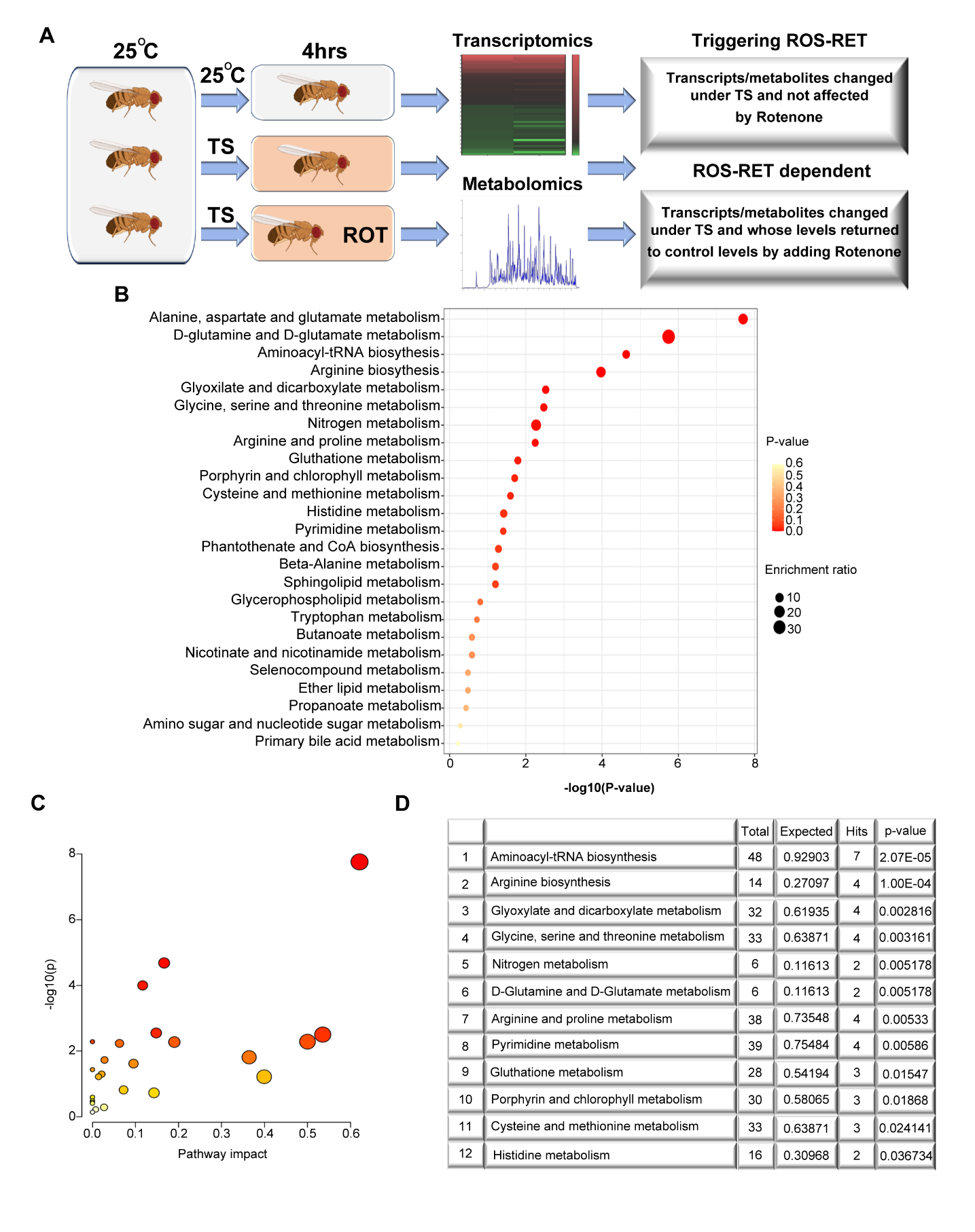
**

**
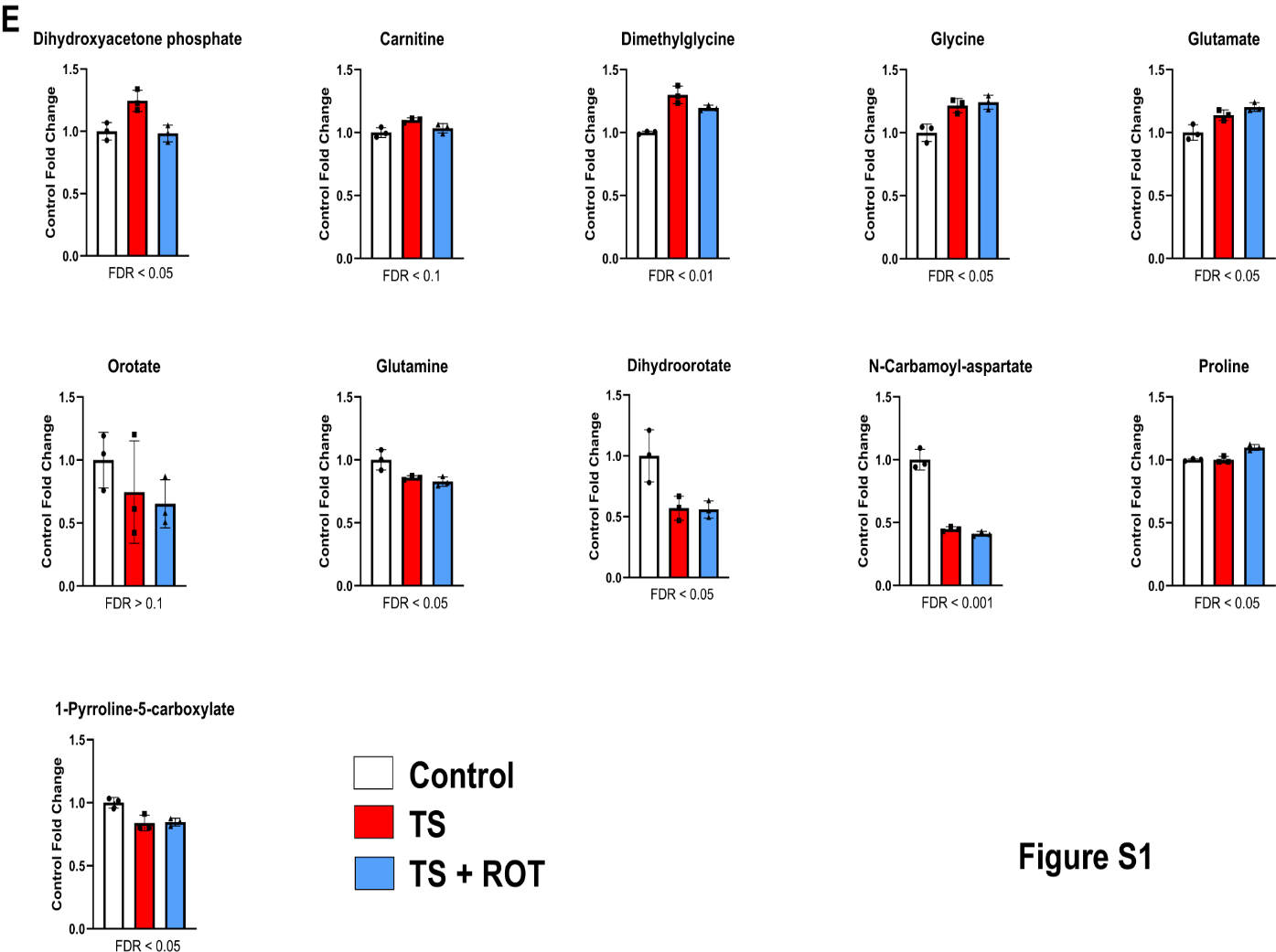
**

**Figure S1.** Linked to Figure 1. (A) Scheme depicts experimental design. We used three different groups: Control (flies that remained at 25°C), TS (flies exposed to 32°C for 4 hours) and TS+ROT (TS flies fed with rotenone to prevent ROS-RET production). Metabolites which trigger ROS-RET: metabolites that were significantly changed in TS and TS+ROT conditions and show the same trend in both groups. Metabolites regulated by ROS-RET: metabolites that were significantly changed in TS and TS+ROT but trends were reversed. (B) Enrichment analysis of metabolites stimulating ROS-RET. (C-D) Pathway analysis of metabolites triggering ROS-RET. (E) Bar plots showing Fold Change of the metabolites represented in the heat map of Figure 1D normalised to control (Control Fold Change). Data is represented as mean ± SEM, N = 3 per experimental group.

**
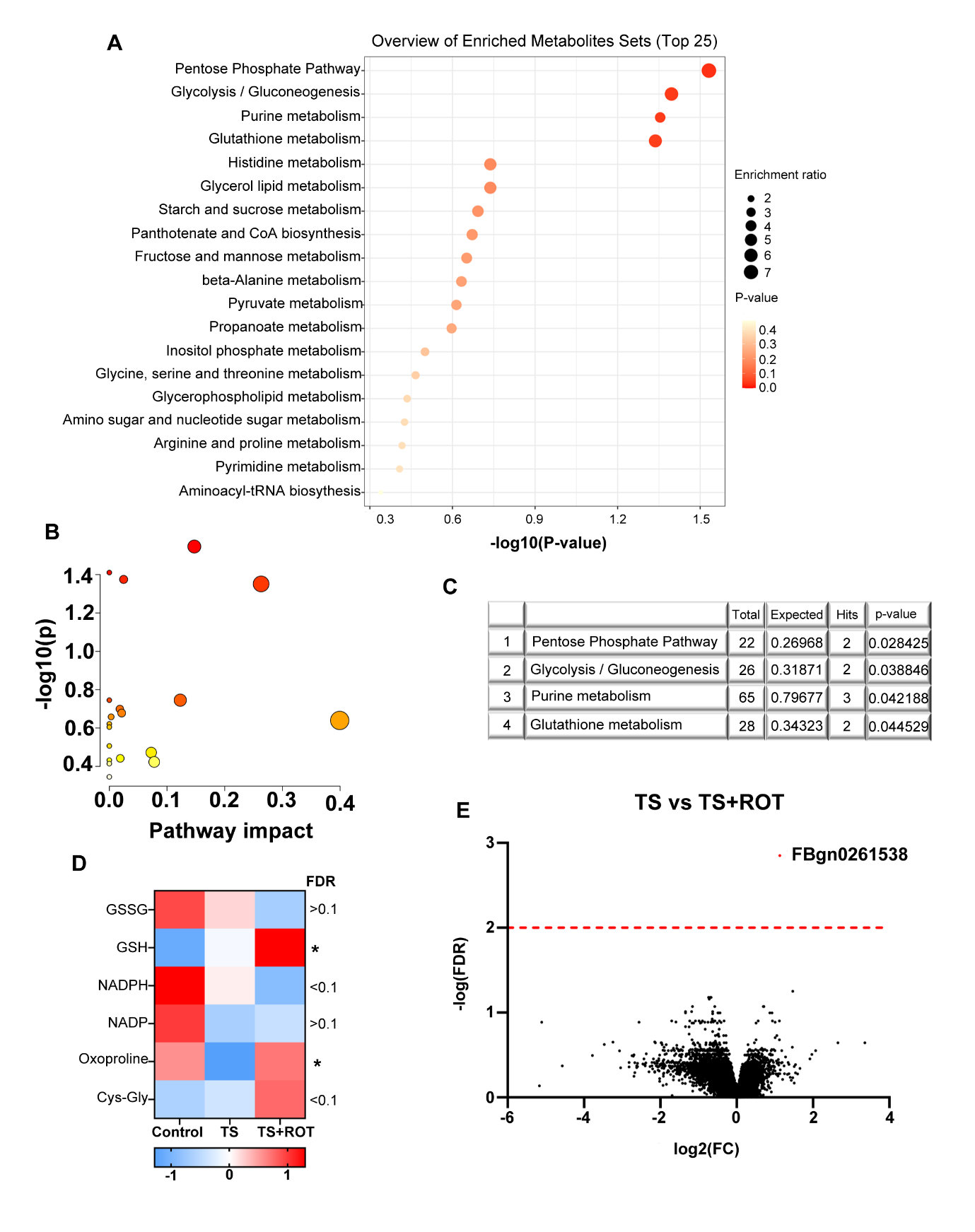
**

**
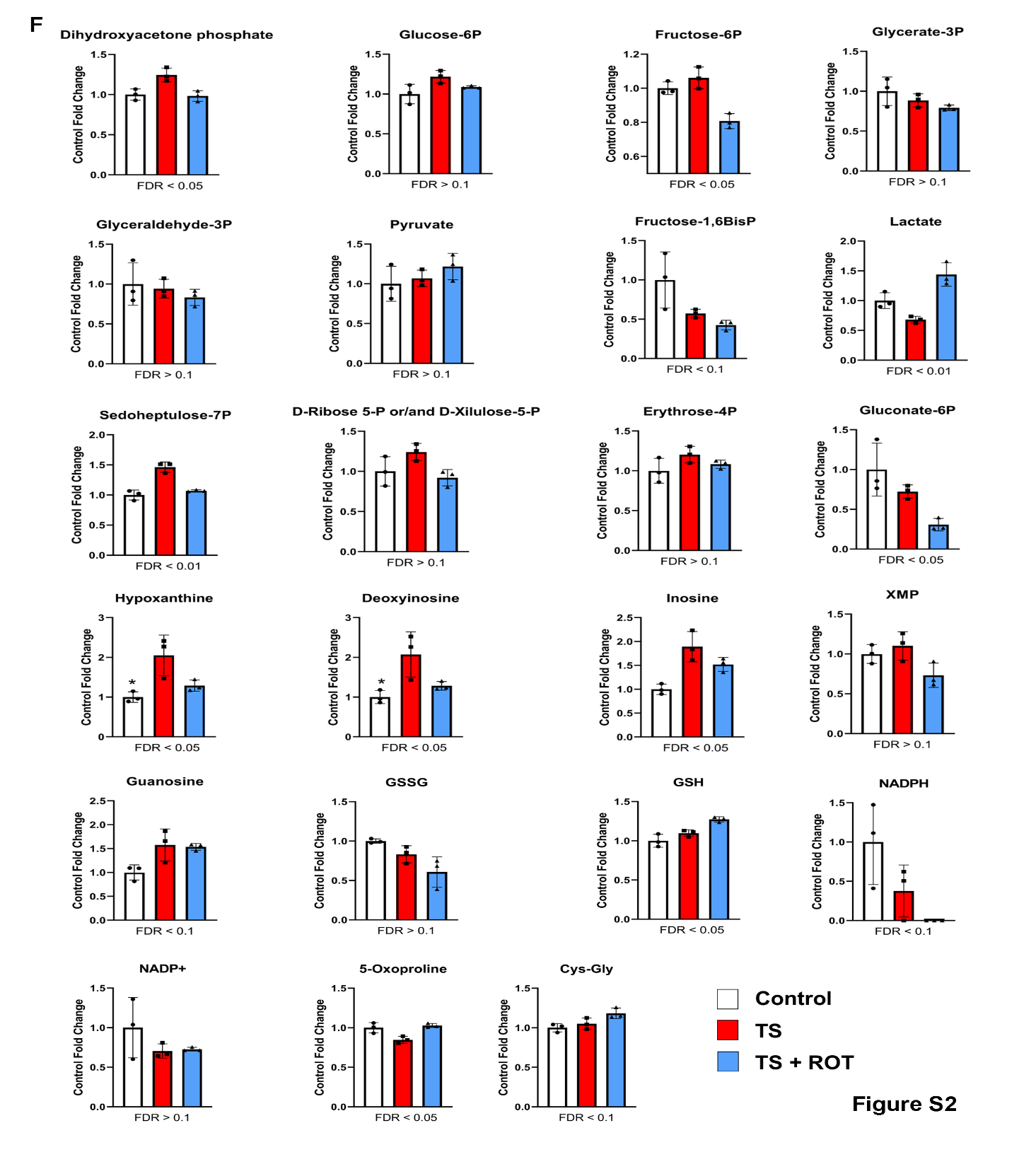
**

**Figure S2.** Linked to Figure 2. (A) Metabolite set enrichment analysis of those metabolites altered by ROS-RET signalling. (B-C) Pathway analysis of metabolites altered by ROS-RET signalling. (D) Heat maps showing metabolites involved in glutathione metabolism identified in the brains of control and TS flies with (TS+ROT) and without rotenone (TS), NS = not significant, *p<0.05, **p<0.01. (E) Volcano plot showing differences in gene expression between TS flies in the absence (TS) or presence of rotenone (TS+ROT). Only one transcript was significantly upregulated in response to ROS-RET (indicated in red). (F) Bar plots showing Fold Change normalised to control of the metabolites represented in the heat maps in Figure 2B-D and panel D of this figure. Data is represented as mean ± SEM, N = 3 per experimental group, except in E where N = 5.


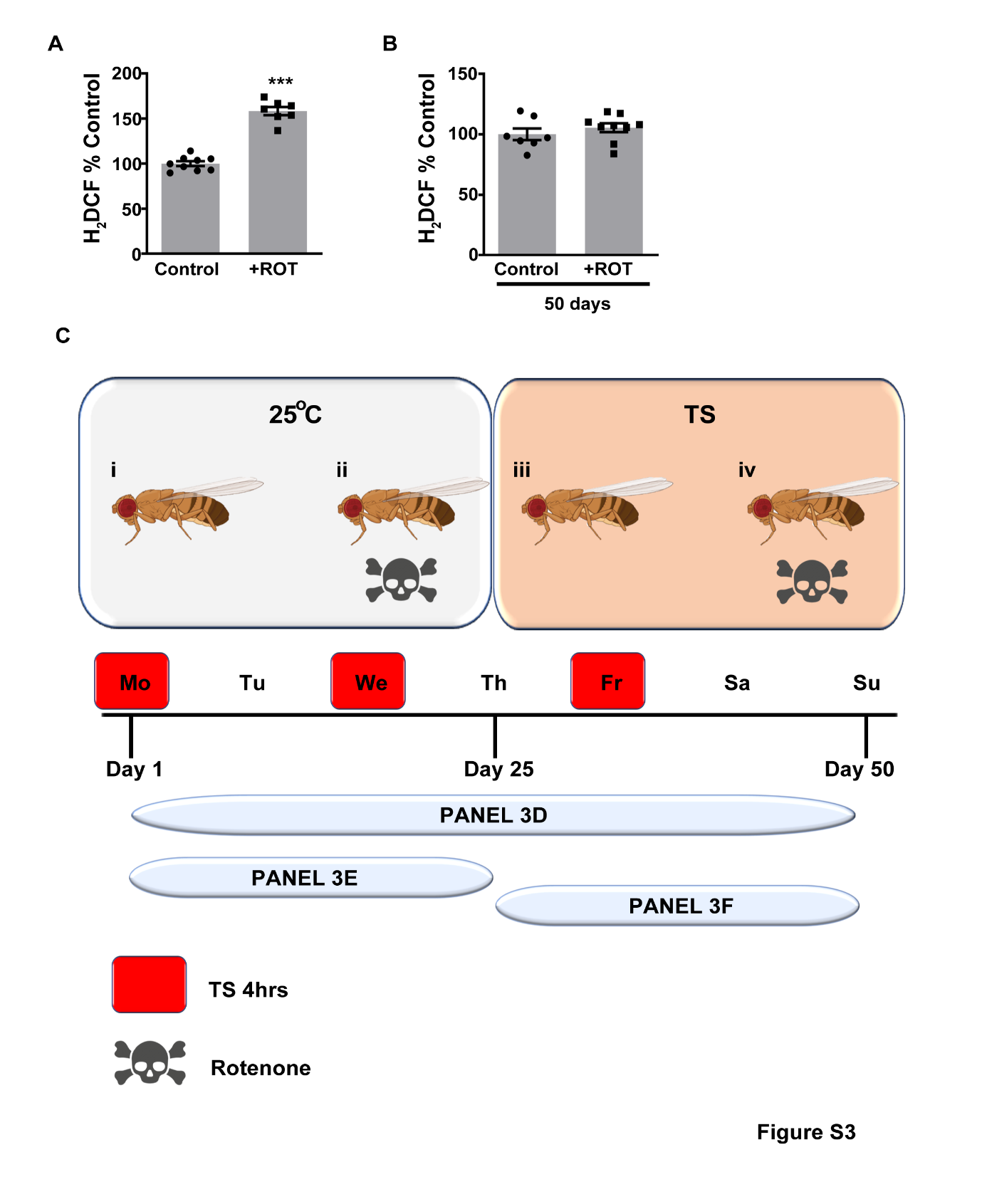


**Figure S3.** Linked to Figure 3. (A) ROS levels in brains of young flies fed with rotenone (+ROT), N = 7-9 per experimental group. (B) ROS levels in brains of old flies fed with rotenone (+ROT), N = 6-7 per experimental group. Data are shown as mean ± SEM. ***p<0.001 (C) Schematic representation of the experimental design indicating experimental conditions. 4 conditions, Control flies kept a 25°C (i), two where flies were exposed to TS for 4 hours three times per week in either the presence (iv) or absence of rotenone (iii) and an additional control group at 25°C which was fed with rotenone 4 hours three times per week (ii). Treatments were administered for either the duration of lifespan (see Panel 3D), from day 1 to day 25 (see Panel 3E), or from day 25 to day 50 (see Panel 3F).


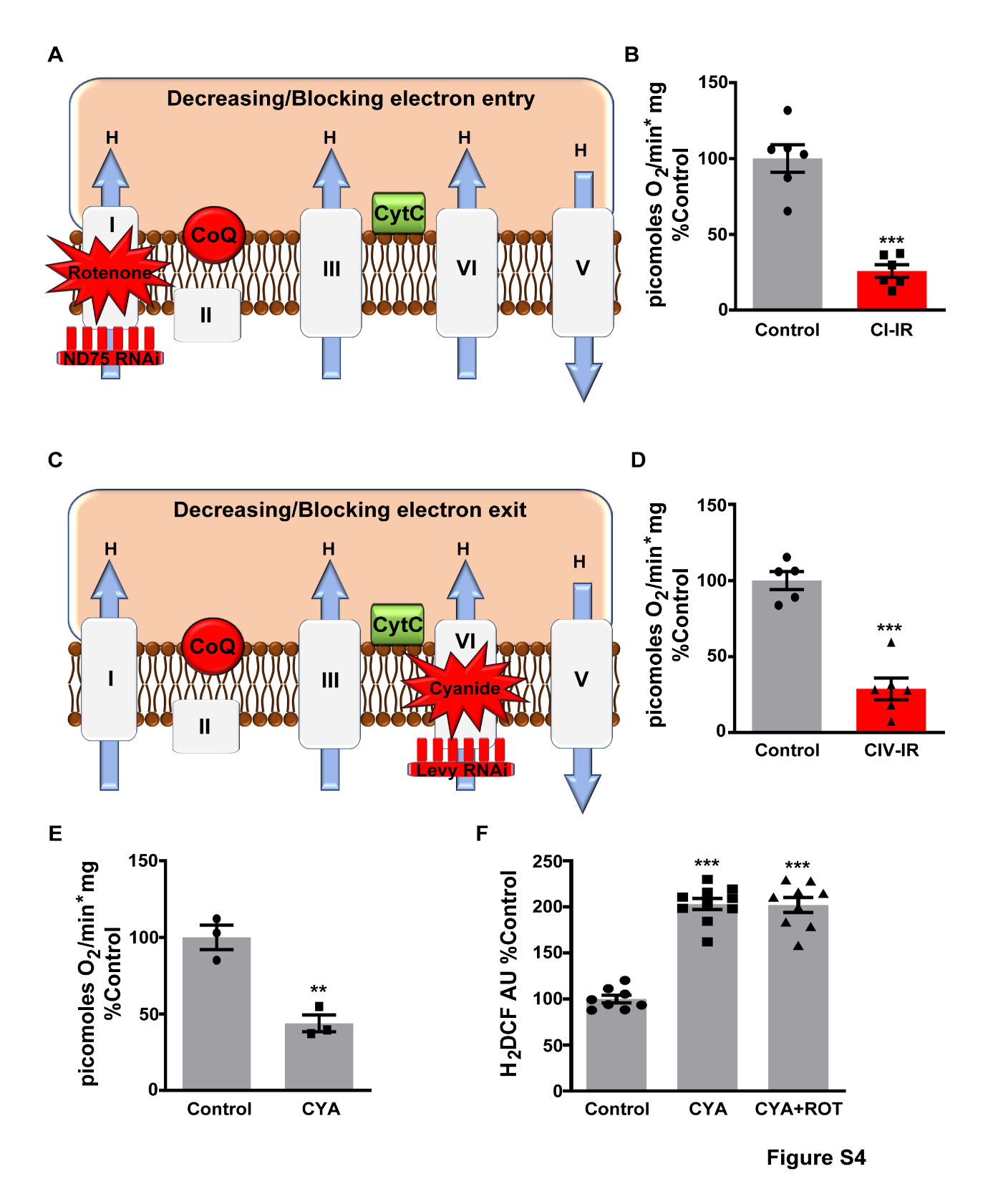


**Figure S4.** Linked to Figure 4. (A) Schematic illustrating strategies used to decrease electron entry into the ETC. (B) Mitochondrial oxygen consumption in homogenates from CI-depleted flies (CI-IR) and controls, N = 6 per experimental group. (C) Schematic diagram with the strategies used to decrease electron exit from the ETC. (D) Mitochondrial oxygen consumption in homogenates from CIV-depleted flies (CIV-IR) and controls, N = 5-6 per experimental group. (E) Mitochondrial oxygen consumption in homogenates of heads from flies fed with cyanide (CYA) and controls, N = 3 per experimental group. (F) ROS levels in brains from control flies, flies fed with cyanide (CYA) and flies fed with cyanide and rotenone (CYA+ROT), N = 8-10 per experimental group. Data are shown as mean ± SEM. **p<0.01, ***p<0.001.
